# NK Cell Activation in Human Hantavirus Infection Explained by Virus-Induced IL-15/IL15Rα Expression

**DOI:** 10.1371/journal.ppat.1004521

**Published:** 2014-11-20

**Authors:** Monika Braun, Niklas K. Björkström, Shawon Gupta, Karin Sundström, Clas Ahlm, Jonas Klingström, Hans-Gustaf Ljunggren

**Affiliations:** 1 Center for Infectious Medicine, Department of Medicine, Karolinska Institutet, Karolinska University Hospital Huddinge, Stockholm, Sweden; 2 Liver Immunology Laboratory, Unit for Gastroenterology and Hepatology, Department of Medicine, Karolinska Institutet, Karolinska University Hospital Huddinge, Stockholm, Sweden; 3 Department of Microbiology, Tumor and Cell Biology, Karolinska Institutet, Stockholm, Sweden; 4 Department of Clinical Microbiology, Infectious Diseases, Umeå University, Umeå, Sweden; Mount Sinai School of Medicine, United States of America

## Abstract

Clinical infection with hantaviruses cause two severe acute diseases, hemorrhagic fever with renal syndrome (HFRS) and hantavirus pulmonary syndrome (HPS). These diseases are characterized by strong immune activation, increased vascular permeability, and up to 50% case-fatality rates. One prominent feature observed in clinical hantavirus infection is rapid expansion of natural killer (NK) cells in peripheral blood of affected individuals. We here describe an unusually high state of activation of such expanding NK cells in the acute phase of clinical Puumala hantavirus infection. Expanding NK cells expressed markedly increased levels of activating NK cell receptors and cytotoxic effector molecules. In search for possible mechanisms behind this NK cell activation, we observed virus-induced IL-15 and IL-15Rα on infected endothelial and epithelial cells. Hantavirus-infected cells were shown to strongly activate NK cells in a cell-cell contact-dependent way, and this response was blocked with anti-IL-15 antibodies. Surprisingly, the strength of the IL-15-dependent NK cell response was such that it led to killing of uninfected endothelial cells despite expression of normal levels of HLA class I. In contrast, hantavirus-infected cells were resistant to NK cell lysis, due to a combination of virus-induced increase in HLA class I expression levels and hantavirus-mediated inhibition of apoptosis induction. In summary, we here describe a possible mechanism explaining the massive NK cell activation and proliferation observed in HFRS patients caused by Puumala hantavirus infection. The results add further insights into mechanisms behind the immunopathogenesis of hantavirus infections in humans and identify new possible targets for intervention.

## Author Summary

Hantaviruses cause severe clinical infections with up to 50% case-fatality rates. The diseases represent an important global health problem as no vaccine or specific treatment is available. The most prominent hallmark in patients is strong immune activation, reflected as massive CD8 T and NK cell expansion, accompanied by severe vascular leakage. The mechanisms behind this massive immune activation are still not fully understood. Here, we first assessed the expression of several activation markers and receptors on NK cells derived from hantavirus-infected patients using flow cytometry. High NK cell activation was observed during the acute phase of clinical infection. To address possible underlying mechanisms explaining this NK cell activation, we established an *in vitro* hantavirus infection model using human primary endothelial cells, the natural *in vivo* targets of the virus. We demonstrate hantavirus-induced IL-15/IL-15Rα on infected endothelial cells, and show that this results in NK cell activation, similar to the profile found in hantavirus-infected patients. Interestingly, these activated NK cells were able to kill uninfected endothelial cells despite their normal expression of HLA class I. The present data add further insights into hantavirus-induced pathogenesis and suggest possible targets for future therapeutical interventions in these severe diseases.

## Introduction

Pathogenic hantaviruses are zoonotic, rodent-borne, viruses that belong to the *Bunyaviridae* family. When infecting humans, they cause hemorrhagic fever with renal syndrome (HFRS) or hantavirus pulmonary syndrome (HPS; also called hantavirus cardio-pulmonary syndrome), two severe acute diseases with case-fatality rates of up to 10% for HFRS and 50% for HPS [1]. HFRS-causing hantaviruses are mainly represented by the prototypic Hantaan virus (HTNV), Puumala virus (PUUV), Dobrava virus, and Seoul virus, whereas HPS-causing viruses include Andes virus, Sin Nombre virus, and related viruses [1].

Hantaviruses can infect several different types of cells, but endothelial and epithelial cells are the primary target cells for hantaviruses in humans [1]. Hantavirus infection of these cells is not cytopathogenic [2]. A common hallmark of HFRS/HPS is, as in other hemorrhagic fevers, increased immune activation and vascular permeability [1]. In the context of immune activation, HFRS and HPS patients have recently been shown to display strong cytotoxic lymphocyte expansions including both NK and CD8 T cells [3]–[6]. Patients also display increased infiltration of immune cells in infected organs, as well as elevated serum levels of, e.g., granzyme B, perforin, and TNF [7]–[10]. However, no overt damage in patients' infected endothelial cells has been observed [11]. Providing some insights into these findings, we recently found hantavirus-infected endothelial cells to be protected from cytotoxic lymphocyte-mediated killing, at least partly, through inhibition of granzyme B and caspase 3 mediated by the hantavirus nucleocapsid protein [12].

NK cells are an important part of the early host defense against virus infections. For instance, humans with specific NK cell-deficiencies often suffer from life threatening virus infections [13]–[15]. The anti-viral response of NK cells includes direct killing of virus-infected cells, mainly mediated through the release of perforin and granzymes, as well as production of pro-inflammatory cytokines including IFN-γ and TNF (reviewed in [13]). These NK cell responses are regulated through a finely tuned balance of signals derived from activating, e.g., NKG2D, and inhibitory NK cell receptors, e.g., killer cell Ig-like receptors (KIRs) and NKG2A/CD94 (reviewed in [16]–[18]). To ensure normal NK cell tolerance to self, and to prevent autoreactivity, most cells in the body express HLA class I ligands for NK cell inhibitory receptors. The expression of KIR and NKG2A inhibitory receptors that recognize self-HLA class I ligands is also needed for NK cells to acquire full functionality, a process referred to as NK cell education, arming, or licensing [19]–[21].

NK cells can be activated by virus-induced cytokines [22]. The principal cytokines involved in NK cell activation are type I interferons (IFN-α/β) as well as IL-12, IL-15, and IL-18 (reviewed in [23]). IL-15 is a pleiotropic cytokine that shares the IL-2 receptor (IL-2R) β and γ chains with IL-2, but has a unique high-affinity IL-15 receptor α chain (IL-15Rα) [24], [25]. IL-15 and IL-15Rα mRNA are abundantly expressed by various immune cell types including monocytes and dendritic cells (DCs), but they can also be expressed in various tissues, e.g., lung, heart, and kidney [24]–[26]. Expression of IL-15 and IL-15Rα is tightly controlled at multiple levels, involving transcription, translation, and intracellular trafficking [27], [28]. Unlike other cytokines, IL-15 is rarely secreted: instead, it is loaded onto IL-15Rα expressed on the same cell and then trans-presented to bystander cells expressing the IL-2Rβ and γ chains [29]. IL-15/IL-15Rα complexes presented to NK cells are essential for NK cell development, differentiation, and survival, and also stimulate NK cell effector functions [30], [31].

The recent observation of a long-term, persistent elevation in NK cell numbers in HFRS patients [3] posed a number of questions that formed the basis for the present study. In particular, (i) how is it associated to states of NK cell activation, (ii) which underlying mechanisms drive the strong NK cell response, and (iii) what potential effects could these NK cells have in hantavirus-infected patients? Here, we first analyzed NK cells in HFRS patients, showing a robust activation of CD56^dim^ NK cells during acute hantavirus infection. This finding prompted us to search for hantavirus-induced mechanism(s) underlying this NK cell activation. Strikingly, *in vitro* studies revealed marked hantavirus-induced IL-15 and IL-15Rα expression on the cell surface of infected endothelial and epithelial cells, and a distinct activation of CD56^dim^ NK cells mediated by trans-presented IL-15. The strength of this NK cell response was such that it led to killing of uninfected endothelial cells despite expression of normal HLA class I levels, while infected cells gained HLA class I expression and resistance to NK cell-mediated apoptosis induction. In summary, these results provide a potential mechanism behind the massive NK cell activation and proliferation observed in clinical HFRS. The consequences of this NK cell activation and proliferation are discussed in the context of human hantavirus immunopathogenesis and implications for future therapeutic strategies.

## Results

### CD56^dim^ NK cells are highly activated in HFRS patients

**Figure 1:**
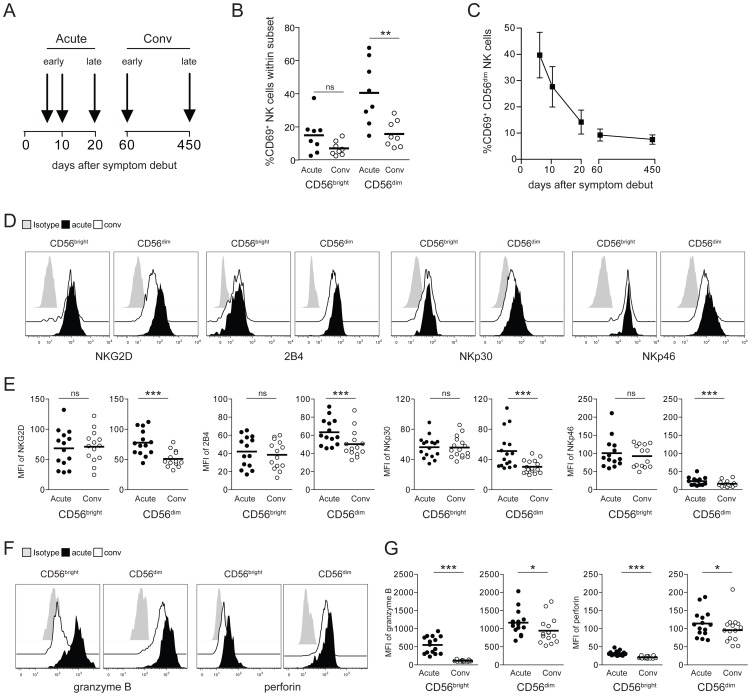
CD56^dim^ NK cells are highly activated in HFRS patients. (A) PBMC from 16 PUUV-infected HFRS patients were collected in the early acute (median d6) and convalescence phase (d60). From 8 HFRS patients additional samples were collected at the indicated time-points and up to day 450. Analyses of NK cell phenotype shown in 1B and 1D–G were performed with samples from patients in the early acute (median d6) and early convalescent phase (d60) of HFRS. (B) Frequencies of CD69-positive CD56^bright^ and CD56^dim^ NK cells in early acute and convalescent (d60) HFRS infection (n = 8). (** p≤0.01, paired *t*-test). (C) Frequencies of CD69-positive CD56^dim^ NK cells from HFRS patients (n = 8) from early acute to convalescence phases are depicted. Values shown are mean (+/− SD). (D and E) Expression levels of activating NK cell receptors on CD56^bright^ and CD56^dim^ NK cells in acute and convalescent phases (D) Representative staining for NKG2D, 2B4, NKp30 and NKp46 on CD56^bright^ and CD56^dim^ NK cells during acute HFRS infection (black) and convalescence (white). Isotype (grey). (E) Expression levels (MFI) of NKG2D, 2B4, NKp30 and NKp46 on CD56^bright^ and CD56^dim^ NK cells in HFRS patients (n = 14–16) in acute (black) and convalescent phases (white). (*** p≤0.001, paired *t*-test). (F and G) Levels of intracellular cytotoxic effector molecules in CD56^bright^ and CD56^dim^ NK cells in acute and convalescent phases. (F) Representative intracellular staining for granzyme B and perforin in CD56^bright^ and CD56^dim^ NK cells in acute HFRS infection (black) and convalescence (white). Isotype (grey). (G) Expression levels (MFI) of intracellular granzyme B and perforin in CD56^bright^ and CD56^dim^ NK cells of HFRS patients (n = 14–16) in acute (black) and convalescent phases of infection (white). (*** p≤0.001, * p≤0.05, paired *t*-test).

Following our previous observation of elevated NK cell numbers in PUUV-infected HFRS patients [3], we here first sought to characterize the activation status of these NK cells. Peripheral blood mononuclear cells (PBMCs) from 16 Swedish HFRS patients were sampled at the time of hospitalization and during the convalescence phase at day 60. For 8 patients, additional samples were available, consecutively collected for a period of up to 450 days (Figure 1A). CD56^bright^ and CD56^dim^ NK cells, the latter constituting the major part of peripheral NK cells [32], were analyzed by flow cytometry for expression of the early activation marker CD69, levels of various activating NK cell receptors, and cytotoxic effector molecules. CD56^dim^ NK cells were shown to express significantly elevated levels of CD69 during acute HFRS with up to 70% of the total CD56^dim^ NK cell population expressing CD69. CD56^bright^ NK cells exhibited some, but clearly lower, levels of activation (Figure 1B, Figure S1A). Percentages of CD69-positive CD56^dim^ NK cells subsequently declined over time (Figure 1C, Figure S1B). During the acute phase of HFRS, CD56^dim^ NK cells also expressed significantly higher levels of the activating NK cell receptors NKG2D and 2B4, and the natural cytotoxicity receptors NKp30 and NKp46 (Figure 1D and E), whereas the expression of other activating receptors including DNAM-1, CD161, and CD16 were not changed over time. In accordance with the relatively lower CD69 induction seen in CD56^bright^ NK cells (Figure 1B, Figure S1A), no marked increase in expression of NK cell activating receptors were observed on these cells (Figure 1D and E). Intracellular levels of the cytotoxic effector molecules granzyme B and perforin were elevated during the acute phase in both, CD56^dim^ and CD56^bright^, NK cell subsets, although expression levels in the CD56^dim^ subset were clearly higher than in the CD56^bright^ NK cells (Figure 1F and G). In summary, cytotoxic CD56^dim^ NK cells were highly activated during the acute phase of clinical HFRS.

### Cell-cell contact with hantavirus-infected endothelial cells results in CD56^dim^ NK cell activation

**Figure 2:**
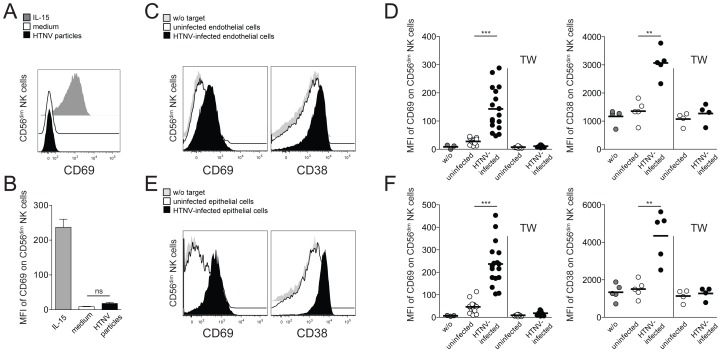
Hantavirus-infected cells mediate contact-dependent activation of CD56^dim^ NK cells. (A) Representative FACS analysis of CD69 expression on CD56^dim^ NK cells after 24 h incubation with HTNV particles (black), IL-15 (grey) or medium (white). (B) Expression level (MFI) of CD69 on CD56^dim^ NK cells (n = 6) treated as indicated. (C–F) CD69 and CD38 expression on CD56^dim^ NK cells after contact with uninfected and HTNV-infected endothelial (C and D) or epithelial (E and F) cells. (C and E) Expression of CD69 and CD38 on CD56^dim^ NK cells incubated with medium (grey), uninfected endothelial or epithelial cells (white) and HTNV-infected endothelial or epithelial cells (black). Representative FACS plots are depicted. (D and F) Summary of CD69 and CD38 expression (MFI) on CD56^dim^ NK cells incubated in the indicated conditions. CD69 (n = 17) and CD38 (n = 5). TW (transwell). (*** p≤0.001, ** p≤0.01, paired *t*-test).

To approach possible mechanisms behind CD56^dim^ NK cell activation in hantavirus-infected patients, we exposed resting primary NK cells from healthy individuals to HTNV and assessed if the virus itself could provoke activation of NK cells. No evidence of hantavirus-replication was observed in HTNV-exposed NK cells, and no CD69 expression was induced on NK cells after exposure to the virus (Figure 2A and B). These findings suggest that NK cells do not respond to direct exposure to hantavirus.

Next, we investigated if NK cells were affected by HTNV-infected primary endothelial and renal epithelial cells, both target cells of hantavirus replication during acute infection in humans. Indeed, CD56^dim^ NK cells co-incubated with HTNV-infected cells acquired an activated phenotype, as reflected by an increase in expression of the activation markers CD69 and CD38 (Figure 2C–F). Activation of CD56^bright^ NK cell was also observed, but to a lower degree when compared to CD56^dim^ NK cells (Figure S2 A and B). No CD56^dim^ NK cell activation was apparent when NK cells and hantavirus-infected cells were separated by transwells (Figure 2D and F), suggesting that CD56^dim^ NK cell activation mediated by hantavirus-infected cells is largely dependent on direct cell-cell contact. Taken together, these results show that HTNV-infected cells can activate NK cells, in particular the CD56^dim^ NK cell subset, in a cell-cell contact-dependent manner.

### Increased expression of IL-15 and IL-15Rα on hantavirus-infected endothelial and epithelial cells leads to CD56^dim^ NK cell activation

Following the results presented above, we sought to identify the mechanism underlying the cell-cell contact-dependent hantavirus-mediated CD56^dim^ NK cell activation. Virus-induced NK cell activation can be mediated by the induction of ligands for various NK cell activating receptors on infected cells. As previously shown for endothelial cells [3], the expression of ligands for the activating NK cell receptors NKG2D and DNAM-1 was not changed by HTNV-infection of renal epithelial cells *in vitro* (Figure S3). These findings shifted our focus to the possible involvement of other molecules likely being responsible for hantavirus-mediated activation of NK cells.

**Figure 3:**
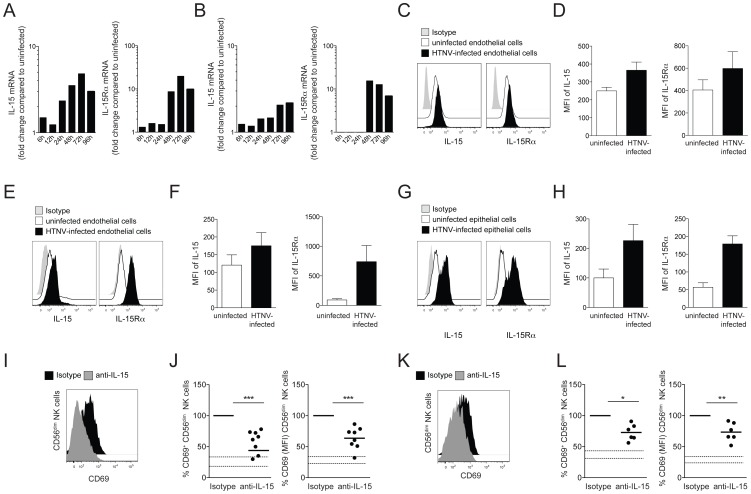
Induced IL-15 and IL-15Rα expression in hantavirus-infected cells drives CD56^dim^ NK cell activation. (A and B) Kinetics of IL-15 and IL-15Rα mRNA-expression in HTNV-infected endothelial (A) and epithelial (B) cells analyzed with RT-qPCR. Fold change compared to the expression level in the respective uninfected cell is depicted. Results of one experiment run in triplicates are shown. (C and D) Expression of IL-15 and IL-15Rα protein in uninfected and HTNV-infected endothelial cells analyzed with flow cytometry after cell permeabilization. (C) One representative FACS analysis is shown. Uninfected cells (white), HTNV-infected cells (black) and isotype control (grey). (D) The expression levels (MFI) of IL-15 and IL-15Rα protein in uninfected (white) or HTNV-infected (black) endothelial cells (n = 3). (E–H) Surface expression of IL-15 and IL-15Rα on uninfected and HTNV-infected endothelial (E and F) and epithelial (G and H). (E and G) Representative FACS analysis of the surface expression of IL-15 and IL-15Rα on uninfected and HTNV-infected endothelial cells and epithelial cells. Uninfected cells (white), HTNV-infected cells (black) and isotype (grey). (F and H) The expression levels (MFI) of IL-15 and IL-15Rα on uninfected (white) or HTNV-infected (black) endothelial (n = 4) and epithelial cells (n = 4) are shown. (I–L) CD69 expression on CD56^dim^ NK cells after co-incubation with HTNV-infected endothelial cells (I and J) and epithelial cells (K and L) in the presence of anti-IL-15 or isotype control antibody. (I and K) Representative FACS analysis of CD69 expression on CD56^dim^ NK cells incubated with endothelial cells (I) and epithelial cells (K) in the presence of isotype control (black) or anti-IL-15 antibody (grey). (J and L) Expression levels (MFI) of CD69 on CD56^dim^ NK cells after co-incubation with endothelial cells (n = 8) (J) and epithelial cells (n = 6) (L). The dashed lines represent upper and lower SEM intervals for means of CD69 expression on CD56^dim^ NK cells after incubation with the respective uninfected cells.

In this context, attention was drawn towards IL-15. IL-15 is essential for NK cell activation, and its expression can be modulated by virus infection of non-immune cells [33], [34]. Strikingly, HTNV-infection was found to up-regulate the expression of IL-15 and IL-15Rα mRNA in both endothelial and epithelial cells (Figure 3A and B). A two-fold increase in IL-15 and ten-fold increase in IL-15Rα mRNA levels were observed 24 h and 48 h, respectively, after HTNV-infection, and IL-15 and IL-15Rα mRNA levels remained at elevated levels until at least 96 h post infection (Figure 3A and B). Since IL-15 expression is subject to complex post-transcriptional control, we assessed the levels of IL-15 and IL-15Rα proteins in infected compared to uninfected cells. HTNV-infection caused elevated levels of total IL-15 and IL-15Rα protein in endothelial cells (Figure 3C and D) and increased cell surface expression of IL-15 and IL-15Rα on infected endothelial and epithelial cells, respectively (Figure 3E–H).

To test if virus-induced IL-15, trans-presented by IL-15Rα, was responsible for hantavirus-mediated CD56^dim^ NK cell activation, an anti-IL-15 neutralizing antibody was added to infected endothelial and epithelial cells to block IL-15 trans-presentation to NK cells. NK cells were co-incubated with hantavirus-infected cells, treated with anti-IL-15 antibody or the respective isotype control, and then assessed for activation, mirrored as CD69 up-regulation using flow cytometry. When this was done, fewer NK cells expressed CD69, and CD69 expression-levels (MFI) were lower on the NK cells expressing CD69 (Figure 3I–L). This results strongly suggest trans-presented IL-15 as a key molecule in inducing CD56^dim^ NK cell activation upon interaction with hantavirus-infected cells.

**Figure 4:**
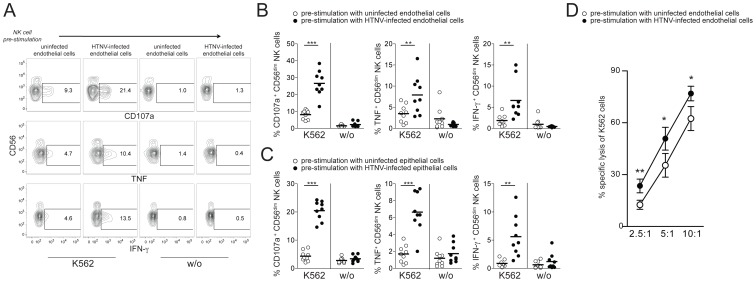
CD56^dim^ NK cells acquire increased functional capacity after contact with hantavirus-infected cells. (A–C) Degranulation (CD107a) and intracellular cytokine production of CD56^dim^ NK cells reacting to K562 cells after pre-stimulation with uninfected or HTNV-infected endothelial and epithelial cells. (A) Representative FACS analysis of CD107a, IFN-γ and TNF expression in one NK cell donor is shown. (B and C) Summary of the CD56^dim^ NK cell responses against K562 cells (n = 9) after pre-stimulation with uninfected (white) or HTNV-infected (black) endothelial (B) and epithelial cells (C). (*** p≤0.001, ** p≤0.01; paired *t*-test). (D) NK cell-mediated specific lysis of K562 cells after pre-stimulation of NK cells with uninfected (white) and HTNV-infected (black) endothelial cells. Depicted are mean values (+/− SD) from 5 donors and 2 independent experiments (** p≤0.01, *p≤0.05; paired *t*-test).

To address the possibility that CD56^dim^ NK cells, activated by hantavirus-infected cells trans-presenting IL-15, gained increased functional capacity, we quantified effector responses against NK cell-sensitive K562 cells. CD56^dim^ NK cells showed significantly increased degranulation, IFN-γ-, and TNF-responses towards K562 cells after pre-stimulation with HTNV-infected cells compared to pre-stimulation with uninfected cells (Figure 4A–C). The elevated levels of CD56^dim^ NK cells degranulation against K562 cells after pre-stimulation with HTNV-infected cells was also associated with an increased specific lysis of K562 cells (Figure 4D). Together, these results identify increased IL-15 and IL-15Rα surface expression on hantavirus-infected endothelial and epithelial cells as important factors for CD56^dim^ NK cell activation and effector functions including cytokine production and cytotoxicity.

### Hantavirus-infected endothelial cells as targets for CD56^dim^ NK cells

**Figure 5:**
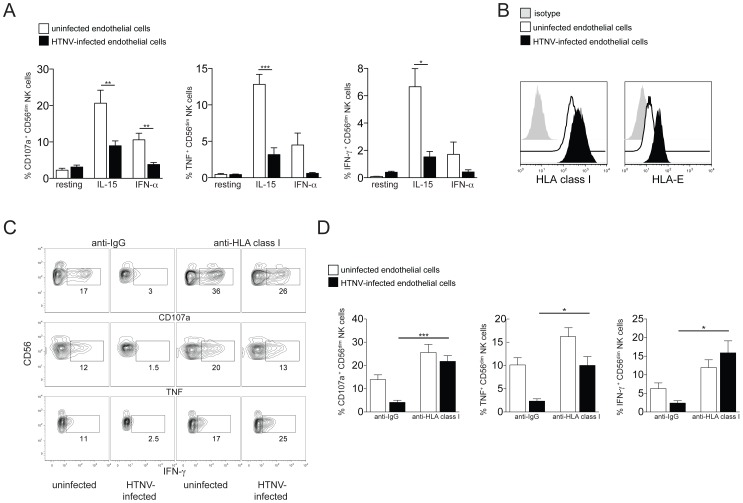
Increased HLA class I expression on hantavirus-infected cells inhibits NK cell effector functions. (A) Degranulation (CD107a) and cytokine production (TNF and IFN-γ) of resting, IL-15 and IFN-α pre-activated CD56^dim^ NK cells reactive against uninfected (white) or HTNV-infected (black) endothelial cells. Results from 2 independent experiments (n = 6) (*** p≤0.001, ** p≤0.01, * p≤0.05; paired *t*-test). (B) Representative staining of HLA class I and HLA-E expression on uninfected (white) or HTNV-infected (black) endothelial cells. Isotype control (grey). (C and D) CD56^dim^ NK cell responses against uninfected and HTNV-infected endothelial cells in the presence of anti-HLA class I or isotype control antibody. (C) Representative FACS analysis of the NK cell responses against uninfected and HTNV-infected endothelial cells in the presence of anti-HLA class I or isotype control antibody is depicted. (D) Frequency of CD56^dim^ NK cells expressing CD107a (n = 6), TNF (n = 3) and IFN-γ (n = 3) in response to uninfected (white) and HTNV-infected (black) endothelial cells in the presence of anti-HLA class I or isotype control antibody (*** p≤0.001, * p≤0.05; paired *t*-test).

Since HTNV-infected endothelial and epithelial cells induced CD56^dim^ NK cell activation and increased their capacity to respond towards K562 target cells, we next sought to assess consequences of interactions between activated NK cells and HTNV-infected endothelial cells. NK cells pre-activated with exogenous IL-15 or IFN-α readily responded to uninfected endothelial cells (Figure 5A). Comparably, responses were weaker towards HTNV-infected endothelial cells (Figure 5A). Similar results were obtained with uninfected and HTNV-infected epithelial cells (Figure S4). As previously reported [3], [35], HTNV-infection of endothelial cells increases their HLA class I expression (Figure 5B). This increase may, at least in part, contribute to weaker effector responses of activated NK cells towards HTNV-infected target cells. Corroborating the latter conclusion, blocking of HLA class I on the infected endothelial cells augmented the functional response of CD56^dim^ NK cells to a level comparable to the response towards HLA class I-blocked uninfected endothelial cells (Figure 5C and D). These results indicate that NK cells are affected by HTNV-infected cells in two contrasting ways: CD56^dim^ NK cells pre-stimulated by HTNV-infected cells are activated with increased cytotoxic potential towards NK cell-sensitive target cells, whereas effector responses against the infected endothelial and epithelial cells themselves are reduced compared to responses towards the uninfected endothelial and epithelial cells.

### CD56^dim^ NK cells activated by hantavirus-infected endothelial cells kill uninfected endothelial cells

**Figure 6:**
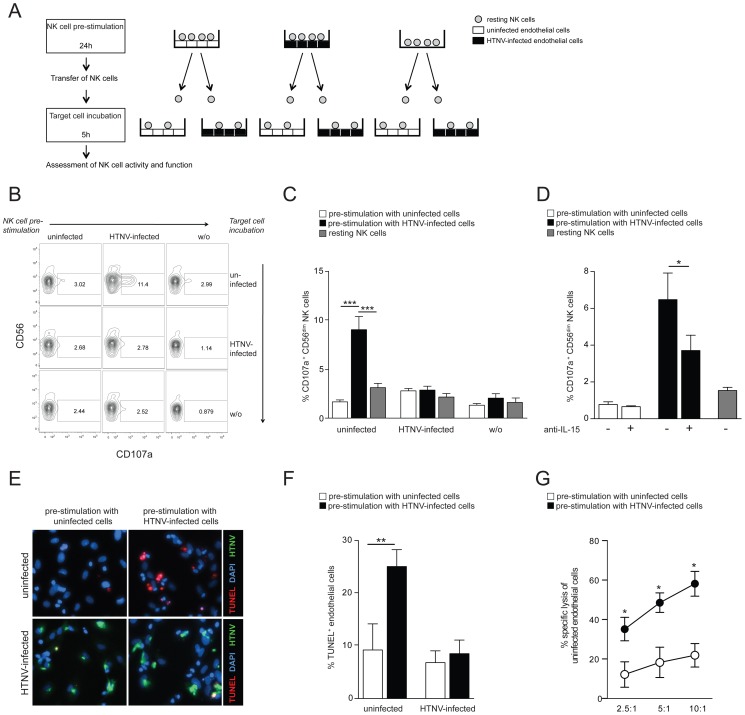
Hantavirus-activated CD56^dim^ NK cells kill uninfected but not hantavirus-infected endothelial cells. (A) Experimental set-up: NK cells were incubated with uninfected or HTNV-infected cells for 24 h then transferred and incubated for another 5 h with uninfected or HTNV-infected cells, followed by assessment of NK cell degranulation and cytotoxicity. (B and C) Degranulation (CD107a) of CD56^dim^ NK cells pre-stimulated with uninfected or HTNV-infected endothelial cells against uninfected and HTNV-infected endothelial cells. (B) FACS analysis of one NK cell donor is shown. (C) CD107a expression on CD56^dim^ NK cells (n = 9) in response to uninfected and HTNV-infected endothelial cells after pre-stimulation with uninfected (white) or HTNV-infected (black) endothelial cells or medium alone (grey). Results from 3 independent experiments (*** p≤0.001; paired *t*-test). (D) Degranulation of CD56^dim^ NK cells (n = 4) against uninfected endothelial cells after pre-stimulation with uninfected (white) and HTNV-infected (black) endothelial cells. When indicated, IL-15 was blocked on endothelial cells during pre-stimulation. Results from 2 independent experiments (* p≤0.05; paired *t*-test). (E and F) Induction of apoptosis in endothelial cells exposed to NK cells pre-stimulated with uninfected or HTNV-infected endothelial cells. (E) One representative immunofluorescent staining is depicted: DAPI (blue), HTNV-nucleocapsid protein (green), TUNEL-positive cells (red). (F) Percentage of TUNEL-positive uninfected and HTNV-infected endothelial cells after exposure to NK cells (n = 6) pre-stimulated with uninfected (white) and HTNV-infected (black) endothelial cells. Results from 3 independent experiments (** p≤0.01; paired *t*-test). (G) NK cell-mediated specific lysis of uninfected endothelial cells after pre-stimulation with uninfected (white) or HTNV-infected (black) endothelial cells. Depicted are mean values (+/− SD) from 5 donors and 2 independent experiments (* p≤0.05).

To finally analyze if NK cell activation mediated by surface-expressed IL-15 on HTNV-infected cells enables them to kill infected or uninfected endothelial cells, we stimulated resting NK cells by incubating them with uninfected and HTNV-infected endothelial cells, respectively. Subsequently, these NK cells were transferred to cultures with uninfected or HTNV-infected cells followed by analysis of NK cell degranulation and NK cell-mediated apoptosis of the target cells (see Figure 6A for experimental outline). CD56^dim^ NK cells pre-stimulated with HTNV-infected endothelial cells degranulated when subsequently co-incubated with uninfected control cells but not with infected cells (Figure 6B and C). Similar results were obtained using primary epithelial cells (Figure S5). Blocking of IL-15 on the infected cells during the pre-stimulation step diminished the following NK cell degranulation against uninfected cells (Figure 6D), showing that HTNV-activated IL-15 expression on infected cells is important for the ability of NK cells to later respond to uninfected endothelial cells. Interestingly, an increased level of apoptosis (Figure 6E and F) and specific lysis (Figure 6G) followed the exposure of uninfected cells to NK cells pre-stimulated with infected cells, suggesting that hantavirus infection may cause NK cell killing of uninfected endothelial cells. Together, these results show that CD56^dim^ NK cells, activated by surface-expressed IL-15 and IL-15Rα on hantavirus-infected cells, overcome HLA class I-mediated self-tolerance and can kill uninfected endothelial cells, whereas HTNV-infected cells are protected from lysis.

## Discussion

We here show that CD56^dim^ NK cells from peripheral blood of hantavirus-infected patients are highly activated in the acute phase of HFRS, as manifested by increased expression of specific activation markers, activating NK cell receptors, and cytotoxic effector molecules. Further studies revealed that CD56^dim^ NK cells, predominantly, can get activated by hantavirus-infected endothelial and epithelial cells, and that these hantavirus-activated NK cells mirrored the NK cell activation profile observed in HFRS patients. IL-15/IL-15Rα cell surface expression on hantavirus-infected endothelial cells was identified as a key factor behind this NK cell activation. Interestingly, infected cells were not killed by NK cells, likely due to increased expression of HLA class I in combination with an inhibition of apoptosis induction in infected cells [12]. The observation that infected cells are not readily killed by NK cells may contribute to the sustained NK cell response observed in clinical HFRS.

In the present study, we observed a relatively stronger activation of CD56^dim^ than of CD56^bright^ NK cells in clinical HFRS. This in contrast to, e.g., acute HCV infection, where it has been shown that both NK cell subsets, CD56^dim^ and CD56^bright^, are activated [36]. This suggests that different viruses may activate specific subsets of NK cells differently. CD56^dim^ NK cells are more prone towards cytotoxicity and respond preferentially to receptor-mediated activation following recognition of target cells, whereas CD56^bright^ NK cells respond primarily to cytokine stimulation by proliferating and producing pro-inflammatory cytokines [37]. Therefore, one could speculate that target cell-dependent activation dominates the shaping of the NK cell response in clinical HFRS. This presumption is supported by our findings that, *in vitro*, hantavirus-infected cells are able to activate NK cells, that this activation is contact-dependent, and that it predominantly affects the CD56^dim^ NK cells (Figure 2 and S2). We identified hantavirus-induced IL-15/IL-15Rα surface expression as one key factor for this NK cell activation (Figure 3). We did not observe infection-dependent changes for known NKG2D and DNAM-1 ligands on infected endothelial and epithelial cells. However, we have previously shown increased expression of the adhesion molecule ICAM-1, a ligand for LFA-1, and HLA-E, a ligand for the activating NKG2C/CD94 NK cell receptor, on HTNV-infected endothelial cells [3]. These ligands might, together with HTNV-induced IL-15, contribute to the activation and proliferation of NK cells observed in HFRS patients.

Corroborating the present findings, levels of IL-15 have been shown to be elevated in sera from patients during acute HFRS infection [3] and in Sin Nombre virus-infected monkeys [38], and an association between IL-15 serum levels and NK cell activation has been reported for other infectious diseases such as acute dengue virus infection [39]. IL-15 can induce increased expression of activating receptors, e.g., NKG2D, NKp30 and NKp46 ([40] and our unpublished observations), suggesting additional cytokine-driven NK cell activation during HFRS. The relatively reduced CD56^bright^ NK cell activation in HFRS patients despite elevated cytokine serum levels might be explained with the specific recruitment of activated CD56^bright^ NK cells to tissue, as suggested for acute influenza virus infection [41]. Corroborating this, no increase in peripheral blood CD56^bright^ NK cell numbers was seen during acute PUUV-infection [3]. Further, IL-15 presented on HTNV-infected endothelial cells might enhance transmigration of NK cells into infected tissue as shown for T cell recruitment into rheumatoid synovial tissue mediated by IL-15 expression on activated endothelial cells [42]. This is supported by findings of elevated numbers of activated T cells and NK cells in endobronchial mucosal biopsies and bronchial lavage fluid from HFRS patients [7].

Hantaviruses cause systemic viremia. We did not detect hantavirus replication in NK cells, and no effect on NK cell activation was observed after exposure of NK cells to hantavirus particles *in vitro*. Together, this indicates that direct infection of NK cells is not needed for NK cell activation in HFRS. Although initially identified as cells capable of reacting spontaneously against transformed cells, it has become apparent that NK cells, in order to perform their optimal effector functions, also undergo a process of priming [43]. During virus infection this can be achieved via DC-presented IL-15/IL-15Rα, an interaction described as the regulatory synapse [44]. DC-mediated NK cell activation enhances NK cell cytotoxicity and cytokine production although activated mature DCs are protected against these NK cell effector functions through high levels of HLA class I [45], [46]. We here show that hantavirus-infected endothelial cells, expressing IL-15/IL-15Rα, stimulate NK cells in a manner reminiscent of this DC-NK cell interaction. The infected endothelial cells are, similar to mature DCs, protected against NK cell attack via elevated HLA class I expression and, as previously reported, further protected against cytotoxic lymphocyte-mediated killing by inhibition of granzyme B and caspase 3 activity [12]. As endothelial cells are in direct contact with blood-derived NK cells, it is tempting to speculate that the endothelial cells, when infected with hantavirus, induce activation of CD56^dim^ NK cells, the dominant NK cell subset in blood. The role of DCs and other accessory cells in NK cell activation during HFRS remains to be investigated. For Lassa virus, an Arenavirus that also causes hemorrhagic fever, infected macrophages, but not DCs, are the main NK cell priming cells *in vitro* [47]. As infection and activation of DCs with hantavirus has been shown *in vitro* [35], it will be of interest to dissect the NK-DC interaction, and possible consequences of this, following hantavirus infection.

Educated NK cells express HLA class I-specific receptors that inhibit NK cell effector functions assuring tolerance to self [19]–[21]. We here show that stimulation of resting NK cells through contact with HTNV-infected endothelial cells can break this NK cell tolerance to self, thereby allowing NK cells to kill uninfected endothelial cells despite their normal HLA class I expression (Figure 6). A more detailed characterization of the HTNV-activated responding NK cells revealed that all CD56^dim^ NK cell subsets, irrespective of their expression of inhibitory HLA class I-specific receptors, were able to degranulate against uninfected cells with comparable strength. The detailed mechanism allowing the NK cells to overcome HLA class I-mediated inhibition remains to be elucidated, but in line with the rheostat model, suggesting that NK cells constantly adapt to their MHC class I environment [48], one could speculate that NK cells adjust to the increased HLA class I expression on HTNV-infected cells, thereby setting a new activation threshold. The lower, but normal, expression of HLA class I on the uninfected cells together with the preserved expression of NKG2D and DNAM-1 ligands potentially triggers NK cell attack and killing. NK cell-mediated killing of autologous non-transformed cells has been shown for immature DCs after priming of NK cells with mature DCs [45], [46], [49].

Whereas NK cells often are crucial for viral elimination, inappropriate NK cell responses can be detrimental and part of virus-associated pathological effects [50], [51]. Endothelial cell destruction, possibly leading to increased vascular permeability, might occur in HFRS patients, as elevated levels of caspase-cleaved cytokeratin-18, a marker of endothelial/epithelial apoptotic cell death, and of cell-free DNA and lactate dehydrogenase have been documented in HFRS patients, indicating increased cell death [52], [53]. Involvement of cytotoxic immune cells in this destruction is indicated by the increased serum levels of perforin and granzyme B found in HFRS patients [8], which is in line with our observation of increased granzyme B and perforin levels in NK cells during the acute phase of HFRS (Figure 1). However, the findings presented here, showing that hantavirus-infected cells inhibit NK cell responses through elevated HLA class I expression, together with our previous study demonstrating the inhibition of granzyme B and caspase 3 activity in infected cells via the hantavirus nucleocapsid protein [12], argue against direct NK cell-mediated killing of hantavirus-infected endothelial cells as a mechanism behind the increased vascular leakage in HFRS. This is corroborated through a study showing no overt damage of hantavirus-infected endothelial cells in deceased patients [11]. A possible alternative mechanism contributing to the increased vascular permeability in HFRS patients is suggested by our demonstration that NK cells, after activation through hantavirus-infected endothelial cells, killed uninfected endothelial cells *in vitro*. NK cell-mediated killing of autologous, bystander CD4 T cells involved in pathologic effects has been suggested during HIV infection [54].

HPS is a severe clinical condition with mortality rates of 30–50% that currently stands with no specific treatment. Previous efforts to treat HPS-patients with antiviral drugs or corticosteroids have not been successful and currently, symptomatic treatment with veno-arterial extracorporeal membrane oxygenation offers the best change for survival when more conventional intensive care therapies have failed [55]. In this regard, it is interesting to note that blocking the effects of IL-15 has been suggested as therapy in other situations where IL-15 is thought to drive the pathology as shown for various autoimmune diseases including rheumatoid arthritis and multiple sclerosis, but also the rare lymphoproliferative T-cell large granular lymphocytic leukemia [56]–[58]. The safety of an humanized anti-IL-2/IL-15Rβ-chain antibody was successfully tested in a phase I clinical trial, supporting the evaluation of this treatment in other IL-15-driven disorders [59]. Thus, it is tempting to speculate that blockade of IL-15 might represent a novel therapeutic target in HPS with the aim to reduce the rapid-onset hyperinflammatory condition these patients present with.

In summary, the present data add further insights into hantavirus-induced pathogenesis by revealing a possible connection between hantavirus-induced IL-15/IL-15Rα expression on infected endothelial cells and the massive NK cell activation observed in HFRS patients. Thus, targeting IL-15 might be useful in the attempt to design specific treatment strategies for this severe disease.

## Materials and Methods

### Blood samples and cells

Blood samples from healthy donors were collected after written informed consent and with the approval from the Regional Ethical Review Board of the Karolinska Institutet, Stockholm, Sweden. PBMC were isolated by density centrifugation (Ficoll-Hypaque; GE Healthcare) followed by negative NK cell isolation (Miltenyi) yielding >98% pure NK cells. PBMC from HFRS patients were collected in CPT tubes (BD Biosciences) after informed consent and approval by the Regional Ethical Review Board of Umeå University, Umeå, Sweden. Acute hantavirus infection was verified from patient plasma as previously described [60]. K562 cells (ATCC CCL-243) were grown in complete RPMI 1640 medium (10% FBS, L-glutamine, penicillin, and streptomycin). Primary human endothelial cells (HUVEC) and renal epithelial cells (Lonza) were grown according to the manufacturer's instructions using EGM-2 BulletKit and REGM BulletKit, respectively.

### HTNV-infection of endothelial/epithelial cells

Prior to infection, cells were seeded in cell culture plates and grown without supplementing the medium with hydrocortisone, until 90% confluency. Propagation and titration of HTNV strain 76–118 was performed on Vero E6 cells as previously described [61]. Cells were infected with MOI 1, resulting in infection rates between 60–80% 72 hours post infection [3].

### Flow cytometry and antibodies

The following monoclonal Abs were used, clone names and conjugates are given within parentheses: pan HLA class I-A647 (W6/32, A647) (Serotech). HLA-E (3D12, PE), IL-15Rα (eBioJM7A4, PE), and CD155 (2H7CD155, PE), (all eBioscience). IL-15 (34559, PE), MICA (159227, APC), MICB (236511, APC), ULBP1 (170818, unconjugated), ULBP2 (165903, unconjugated), ULBP3 (166510, unconjugated), ULBP4 (unconjugated) (all R&D). CD107a (H4A3, PE and FITC), IFN-γ (25723.11, FITC), granzyme B (GB11, AF647), perforin (δG9, FITC), CD14 (MφP9, APC-Cy7 and V500), CD19 (HIB19, APC-Cy7 and V500), CD16 (3G8, Pacific Blue), CD56 (B159, PE-Cy7), CD69 (FN50, APC and APC-Cy7), DNAM-1 (Dx11, FITC), NKG2D (1D11, PE), and CD112 (R2.525, PE) (all BD Biosciences). TNF-α (MAb11, Pacific blue), CD107a (H4A3, Brilliant Violet 421), CD56 (HCD56, Brilliant Violet 711), and CD38 (HIT2, AF700), (all BioLegend). NKG2A (Z199, APC), CD56 (NHK-1, ECD), CD69 (TP1.55.3, ECD), CD3 (UCHT1, PE-Cy5), 2B4 (C1.7, PE), NKp30 (Z25, PE and APC), and NKp46 (BAB281, PE), (all Beckman Coulter). CD3 (UCHT1, Cascade Yellow), (DakoCytomation). Unconjugated antibodies were detected using an anti-IgG secondary antibody (X56, APC, BD Biosciences). Live/Dead Aqua (Invitrogen) was used to exclude dead cells. Briefly, cells were stained for 20 min at 4°C in FACS buffer (PBS 2% FBS, 2mM EDTA) and fixed in 1% PFA. For intracellular staining, cells were stained for surface markers and then fixed and permeabilized using the Cytofix/Cytoperm kit (BD Biosciences). Samples were acquired on a CyAn ADP instrument (Beckman Coulter) or a LSRFortessa (BD Biosciences) and analyzed using FlowJo software version 9.7.6 (Tree Star Inc).

### Functional NK cell assays

Prior to use in functional assays, isolated primary NK cells were pre-stimulated with IL-15 (20 ng/ml, R&D) or IFN-α (500 U/ml) overnight, or co-incubated with uninfected or HTNV-infected endothelial and epithelial cells at an effector/target ratio of 1∶1 at 37°C in complete RPMI medium. In some experiments IL-15 surface-expression on HTNV-infected endothelial cells was blocked using a neutralizing IL-15 mAb (R&D, clone 34593). For determination of NK cell effector functions, NK cells were incubated with respective target cells at an effector/target ratio of 1∶1 for 6 hours in the presence of anti-CD107a (H4A3, FITC, BD Biosciences). Brefeldin A and monensin (BD Biosciences) were added for the last 5 hours of the assay. If indicated, HLA class I was blocked on the endothelial cells using a combination of A6–136 hybridoma (kindly provided by Dr D Pende, Istituto Nazionale per la Ricerca sul Cancro, Genoa, Italy) and Dx17 mAbs (BD Biosciences) for 30 minutes at room temperature. Cytotoxicity assays were performed by labelling K562 and endothelial cells with CFSE (Invitrogen) and co-culturing these cells with NK cells, pre-stimulated as described above, at the indicated effector/target ratios for 4 hours. Cells were subsequently stained with Live/Dead Fixable Dead cell stain (Invitrogen). Cytotoxicity was calculated as followed: (% dead target cells in co-culture with NK cells−% dead target cells alone)/(100%−% dead target cells alone).

### TUNEL assay

Apoptosis was assessed 5 h after transfer of NK cells to endothelial cells grown on glass slides with TUNEL staining. TUNEL assay (Roche) was performed as previously described [62]. After TUNEL reaction, cells were subjected to staining with anti-nucleocapsid protein mAb, followed by FITC-conjugated goat anti-mouse IgG (Sigma-Aldrich). Nuclei were counter stained with DAPI (Sigma-Aldrich).

### Quantitative RT-PCR

Total RNA was isolated using TriPure Isolation Reagent (Roche Applied Science) and treated with Turbo DNA-free (Ambion) to remove any contaminating DNA. cDNA synthesis was performed using Superscript™ III Reverse Transcriptase, random primers, and RNAseOUT™ Recombinant Ribonuclease Inhibitor (Life Technologies). Total RNA was measured with TaqMan Gene Expression Assay for human IL-15, IL-15Rα and β-actin (all Life Technologies). The quantitative real-time PCR was performed using ABI7900 HT Fast sequence detection system (Life Technologies). Data were analysed with SDS software version 2.3. Data were normalized to β-actin and presented as the change in induction relative to that of uninfected cells.

### Statistical methods

For comparison of matched groups the paired *t*-test (normally distributed data) was performed using GraphPad Prism software version 5.0 (GraphPad Software Inc.). Asterisks denote statistical significance (_*_ p<0.05, _**_ p<0.01, _***_ p<0.001).

### Ethics statement

Blood samples from healthy donors were collected after written informed consent and with the approval from the Regional Ethical Review Board of the Karolinska Institutet, Stockholm, Sweden.

## Supporting Information

Figure S1 **Predominant activation of the CD56^dim^ NK cell subset in acute HFRS.** (A) Representative FACS analysis of the CD69 expression on CD56^bright^ and CD56^dim^ NK cells of one HFRS patient in acute (black) and convalescent phase (white) of HFRS. (B) FACS analysis of the CD69 expression on CD56^dim^ NK cells (n = 8) analyzed at 4 consecutive timepoints in acute and convalescent phases of HFRS in 8 patients (P1–P8).
(PDF)

Figure S2 **Pronounced activation of CD56^dim^ NK cells following contact with HTNV-infected cells.** (A) Representative FACS analysis of the CD69 expression on CD56^bright^ and CD56^dim^ NK cells, defined by their CD56 and CD16 expression, after 24 h pre-stimulation with uninfected (white) and HTNV-infected (black) endothelial cells or medium alone (grey). Expression levels (MFI) are indicated. (B) Summary of the expression levels (MFI) of CD69 on CD56^bright^ and CD56^dim^ NK cells after pre-stimulation with uninfected (white) and HTNV-infected (black) endothelial and epithelial cells (n = 17) or on resting NK cells (n = 8). Data from 6 independent experiments are shown (*** p≤0.001; paired *t*-test).
(PDF)

Figure S3 **Expression of activating NK cell receptor ligands on primary renal epithelial cells is not modulated by HTNV infection.** FACS analysis showing the expression of the ligands for the activating NK cell receptors NKG2D (ULBP-1-4 and MICA/B) and DNAM-1 (CD155 and CD112) on primary renal epithelial cells. Isotype control (grey), uninfected (white) and HTNV-infected (black). One representative FACS staining out of 4 is shown.
(PDF)

Figure S4 **HTNV infection of renal epithelial cells inhibits CD56^dim^ NK cell effector responses.** HTNV infection of primary renal epithelial cells inhibits degranulation (CD107a) and cytokine production (TNF and IFN-γ) in IL-15 pre-activated primary NK cells (n = 6). Data are from 2 independent experiments (*** p≤0.001, ** p≤0.01, * p≤0.05; paired *t*-test).
(PDF)

Figure S5 **CD56^dim^ NK cell activation** ***via*** **contact with HTNV-infected epithelial cells enables increased degranulation against uninfected epithelial cells.** Degranulation (CD107a) of CD56^dim^ NK cells (n = 9), pre-stimulated with uninfected (white) and HTNV-infected (black) epithelial cells or medium alone (grey), against uninfected and HTNV-infected epithelial cells was assessed using flow cytometry. Results are from 3 independent experiments. (*** p≤0.001; paired *t*-test).
(PDF)
