# Supplementary figures and images for: NK Cell Activation in Human Hantavirus Infection Explained by Virus-Induced IL-15/IL15Rα Expression
Source: PLoS Pathog. 2014 Nov 20;10(11):e1004521. doi: 10.1371/journal.ppat.1004521 (PMC4239055; doi:10.1371/journal.ppat.1004521)

A

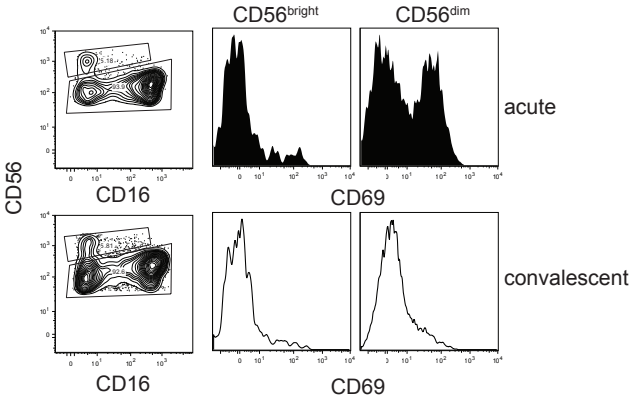

B

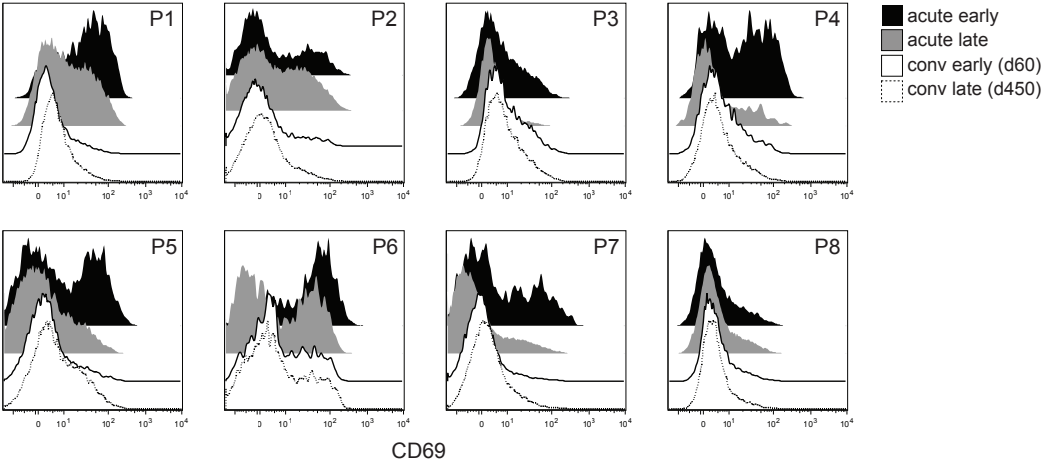

Supplement: Figure S1 — Predominant activation of the CD56dim NK cell subset in acute HFRS. (A) Representative FACS analysis of the CD69 expression on CD56bright and CD56dim NK cells of one HFRS patient in acute (black) and convalescent phase (white) of HFRS. (B) FACS analysis of the CD69 expression on CD56dim NK cells (n = 8) analyzed at 4 consecutive timepoints in acute and convalescent phases of HFRS in 8 patients (P1–P8). (PDF) [file ppat.1004521.s001.pdf]

A

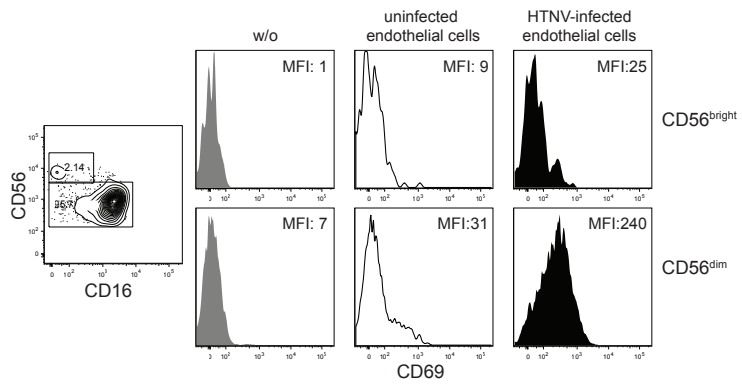

B

- medium
- uninfected cells
- HTNV-infected cells

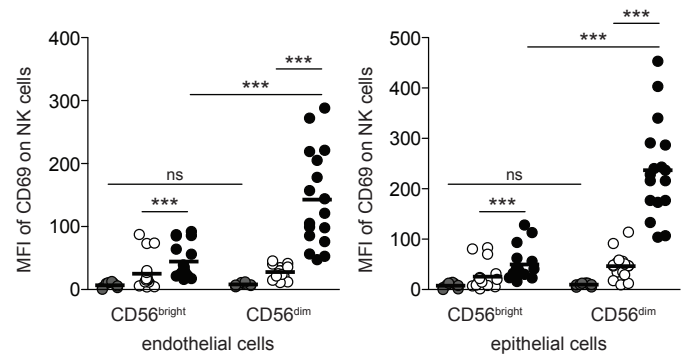

Supplement: Figure S2 — Pronounced activation of CD56dim NK cells following contact with HTNV-infected cells. (A) Representative FACS analysis of the CD69 expression on CD56bright and CD56dim NK cells, defined by their CD56 and CD16 expression, after 24 h pre-stimulation with uninfected (white) and HTNV-infected (black) endothelial cells or medium alone (grey). Expression levels (MFI) are indicated. (B) Summary of the expression levels (MFI) of CD69 on CD56bright and CD56dim NK cells after pre-stimulation with uninfected (white) and HTNV-infected (black) endothelial and epithelial cells (n = 17) or on resting NK cells (n = 8). Data from 6 independent experiments are shown (*** p≤0.001; paired t-test). (PDF) [file ppat.1004521.s002.pdf]

Isotype  
uninfected  
HTNV-infected

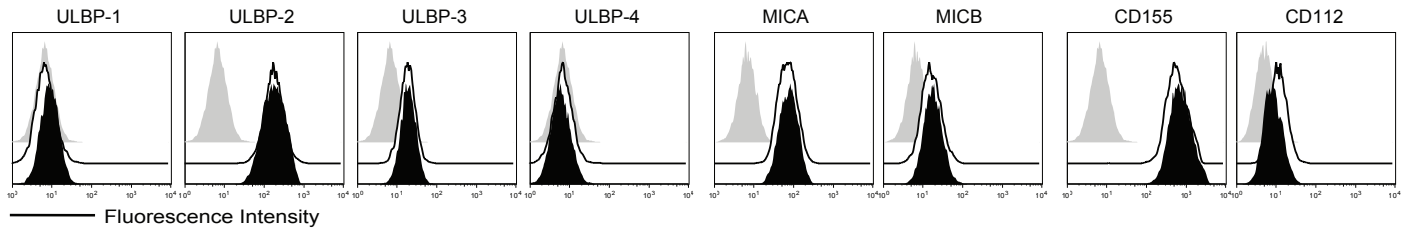

Supplement: Figure S3 — Expression of activating NK cell receptor ligands on primary renal epithelial cells is not modulated by HTNV infection. FACS analysis showing the expression of the ligands for the activating NK cell receptors NKG2D (ULBP-1-4 and MICA/B) and DNAM-1 (CD155 and CD112) on primary renal epithelial cells. Isotype control (grey), uninfected (white) and HTNV-infected (black). One representative FACS staining out of 4 is shown. (PDF) [file ppat.1004521.s003.pdf]

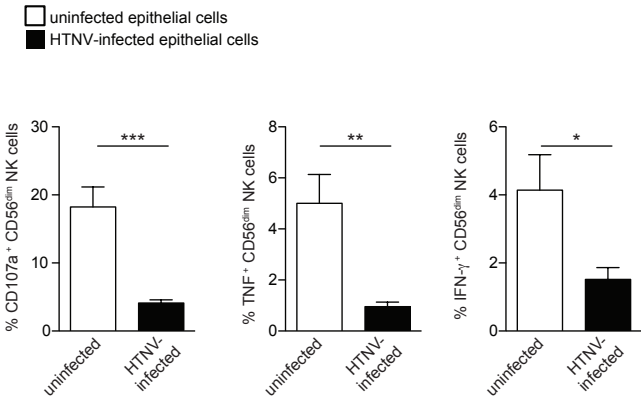

Supplement: Figure S4 — HTNV infection of renal epithelial cells inhibits CD56dim NK cell effector responses. HTNV infection of primary renal epithelial cells inhibits degranulation (CD107a) and cytokine production (TNF and IFN-γ) in IL-15 pre-activated primary NK cells (n = 6). Data are from 2 independent experiments (*** p≤0.001, ** p≤0.01, * p≤0.05; paired t-test). (PDF) [file ppat.1004521.s004.pdf]

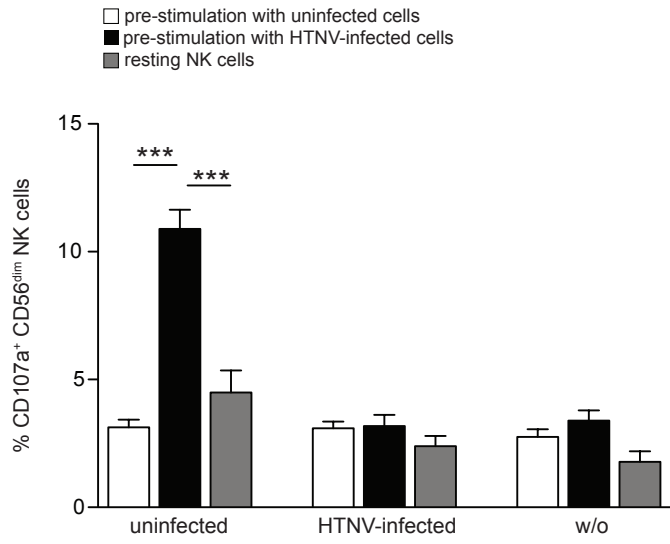

Supplement: Figure S5 — CD56dim NK cell activation via contact with HTNV-infected epithelial cells enables increased degranulation against uninfected epithelial cells. Degranulation (CD107a) of CD56dim NK cells (n = 9), pre-stimulated with uninfected (white) and HTNV-infected (black) epithelial cells or medium alone (grey), against uninfected and HTNV-infected epithelial cells was assessed using flow cytometry. Results are from 3 independent experiments. (*** p≤0.001; paired t-test). (PDF) [file ppat.1004521.s005.pdf]
